# Chemical Constituents and Their Bioactivities of Plants from the Genus *Eupatorium* (2015–Present)

**DOI:** 10.3390/biology13050288

**Published:** 2024-04-24

**Authors:** Hao Geng

**Affiliations:** School of Science, Xichang University, Xichang 615000, China; genghao@xcc.edu.cn or genghao1709@163.com

**Keywords:** natural products, plant-derived natural products, *Eupatorium*, chemical constituents, biological activities

## Abstract

**Simple Summary:**

Based on the important findings of our research group about the chemical constituents of *Eupatorium adenophorum*, the present review shares an update about the research progress on the chemical constituents of *Eupatorium* and their biological activities in the last 10 years. For the first time, it also reviews some studies investigating the chemical constituents of the plant. Considering the multiple properties of this genus, the next step should be to strengthen the study of the action mechanism underlying the active components of this genus. Hopefully, this review can provide new insights for prompting future research on *Eupatorium* applications and drug development.

**Abstract:**

The genus *Eupatorium* belongs to the Asteraceae (Compositae) family and has multiple properties, such as invasiveness and toxicity, and is used in folk medicine. The last review on the chemical constituents of this genus and their biological activities was published in 2015. The present review provides an overview of 192 natural products discovered from 2015 to the present. These products include 63 sesquiterpenoids, 53 benzofuran derivatives, 39 thymol derivatives, 15 fatty acids, 7 diterpenoids, 5 monoterpenoids, 4 acetophenones, and 6 other compounds. We also characterized their respective chemical structures and cytotoxic, antifungal, insecticidal, antibacterial, anti-inflammatory, and antinociceptive activities.

## 1. Introduction

Plant-derived natural products have always been a paramount source of novel drugs and pesticides [1,2,3,4,5,6,7,8]. For example, the plant-derived drugs paclitaxel (Taxol) and artemisinin are widely used in antitumor and antimalarial treatment, respectively, and continue to occupy a crucial position among other drugs used for these medical conditions [9,10,11,12,13,14]. Meanwhile, active plant-derived natural products can also serve as substrates for structural modifications for new drug discovery. For example, the anticancer drugs topotecan and irinotecan are the derivatives of camptothecin, which is isolated and identified from the plant *Camptotheca acuminata* [15,16].

**Figure 1:**
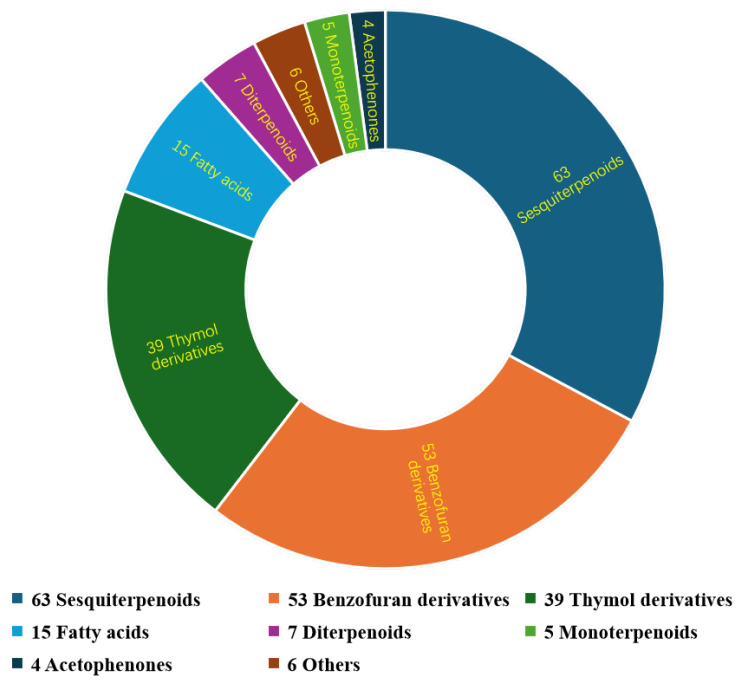
Classification and proportion of the reviewed natural products from *Eupatorium*.

*Eupatorium* is a large genus belonging to the Asteraceae family that contains approximately 1200 species. This genus is widely distributed in global countries, such as America, Europe, Africa, and Asia [17]. The chemical constituents of *Eupatorium* have been investigated for more than 100 years, starting from the study of the volatile oil constituents of E. triplinerve [18]. Until now, more than 300 compounds have been reported to be present in *Eupatorium*, of which some have exhibited certain anticancer, antibacterial, and anti-inflammatory effects [19,20]. Among them, flavonoids and terpenes are the two main chemical constituents of *Eupatorium*. However, the latest reviews discussing the phytochemical investigations and the biological activities of this genus were published almost 10 years ago [20]. Recent major progress in the study of the chemical constituents of *E. adenophorum* was made by our group. We discovered two classes of sesquiterpenoids with novel structures, which were continuously selected as hot molecules by Natural Product Reports (NPRs) [21,22]. Considering that *E. adenophorum* has a potent affinity toward the other plants of the genus, we believe that the discovery of novel structural and active chemical components in the genus *Eupatorium* deserves further investigation. Consequently, to attract more research attention toward this genus, we summarized the research progress of natural products of this genus discovered since 2015, including their sources, structure types, and biological activities. Here, we reviewed a total of 192 compounds (Figure 1), including their chemical structures and biological activities. In the framework of this review presentation, we want to classify those natural products based on the plant species that produce them, rather than their structural types. We hope this review provides insights into the in-depth study, development, and utilization of this genus.

## 2. Progress on Chemical Components and Their Biological Activities of the Genus *Eupatorium*

### 2.1. Chemical Components of E. adenophorum and Their Biological Activities

**Figure 2:**
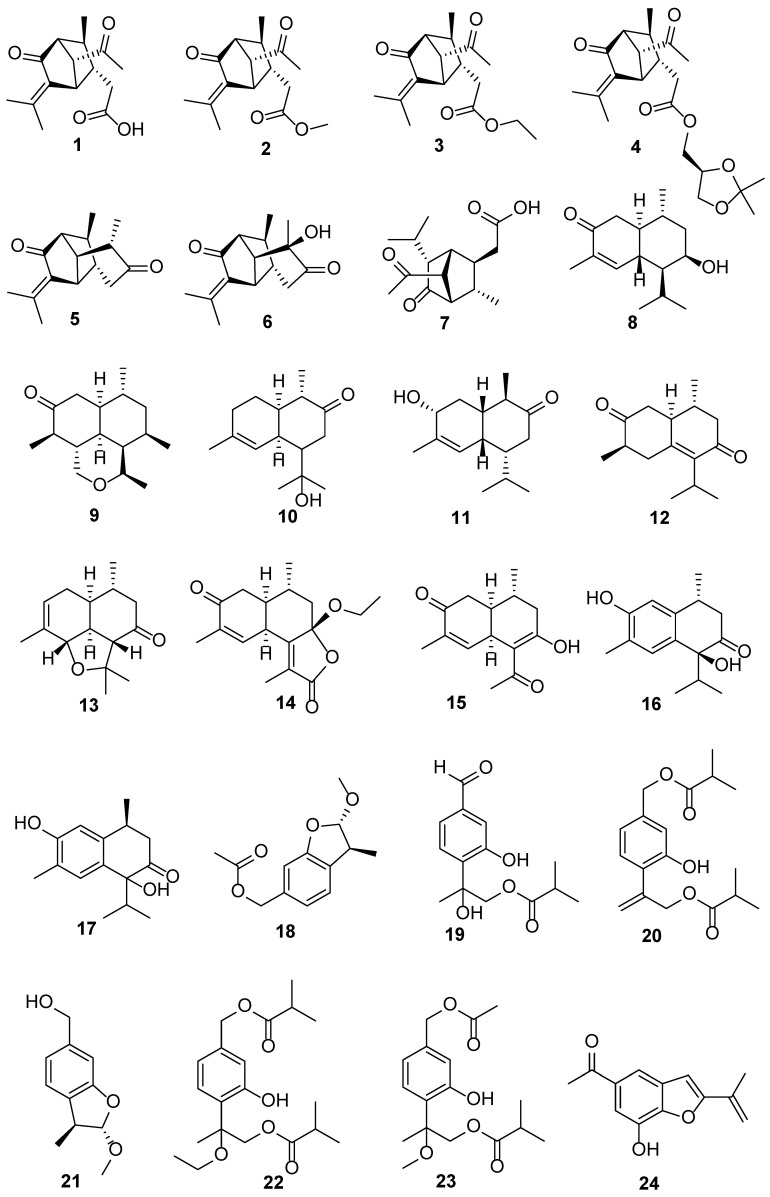

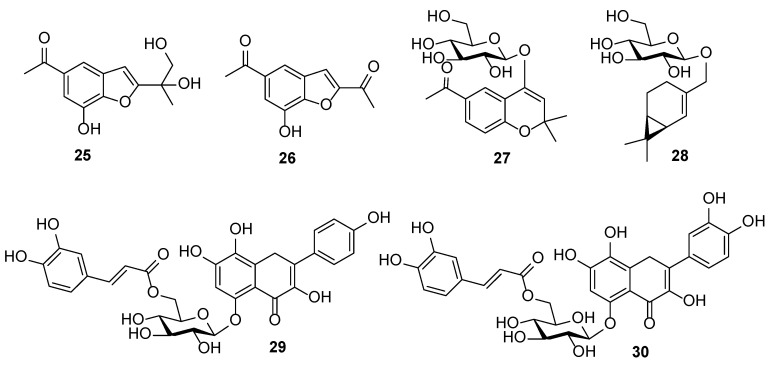
The chemical structures isolated from *E. adenophorum*.

*E. adenophorum* Spreng. (*E. adenophorum*) is synonymous with *Ageratina adenophora* (Spreng.) R. M. King & H. Rob., a perennial and herbaceous invasive plant that is ubiquitous worldwide [23]. Although it is invasive, it has been traditionally used as a medicine for treating wounds, inflammation, fever, diabetes, dysentery, and other ailments. Phytochemical investigations have revealed that this is a sesquiterpenoid-rich plant (Figure 2). In total, 30 new compounds were reported (Table 1), namely 17 sesquiterpenoids, 6 thymol derivatives, 3 benzofuran derivatives, 2 flavonoid glycosides, 1 monoterpenoid glucoside, and 1 chromene derivative [23,24,25,26,27,28,29,30,31,32,33,34,35].

Compounds **1**–**7** represent two classes of sesquiterpenoids with a novel carbon skeleton. Eupatorid A (**1**) and its esterified derivatives, eupatoresters A–C (**2**–**4**) [23] and dihyroeupatorid A (**7**) [26], have a 5/5 bicyclic carbon skeleton. Adenophorone (**5**) [28] and eupatorione A (**6**) [25] possess a 5/5/6 tricyclic carbon skeleton. Conspicuously, *NPRs* had continuously selected compounds **1** and **6** as hot molecules [21,22], because their structures were novel. Unfortunately, the aforementioned seven compounds exhibited no significant activities in the anti-inflammatory, in vitro tumor growth inhibitory, and antibacterial assays, except **5**, which displayed potent neuroprotective activity in H_2_O_2_-treated human neuroblastoma cells (SH-SY5Y) and pheochromocytoma cells (PC12) [24]. Compounds **8**–**17** are typical cadinene-type sesquiterpenoids. However, none of them exhibited significant activities in bacteriostatic, α-glycosidase, and acetylcholine esterase (AChE) inhibitory tests [26,27,28,29,30,31]. Compounds **18**–**23** are thymol derivatives. Compound **18** displayed in vitro bacteriostatic activity against Gram-positive bacteria such as *Staphylococcus aureus*, *Bacillus cereus*, and *B. subtilis*, with minimum inhibitory concentrations ranging from 25 to 50 μg/mL [31,32,33]. Compound **19** exhibited a strong activity against five microorganisms, *S. aureus*, *B. cereus*, *B. thuringiensis*, *Escherichia coli*, and *Salmonella enterica*, with MIC values ranging from 3.9 to 15.6 μg/mL. Additionally, compound **19** showed strong cytotoxicity against human breast cancer cells (MCF-7), human cervical carcinoma cells (HeLa), and human large-cell lung cancer cells (NCI-H460) and its half-maximal inhibitory concentration (IC50) values were 7.45, 9.45, and 8.32 μM, respectively [32]. Compounds **24**–**26** are benzofuran derivatives. Among them, compound **24** at 50 μg/disk exhibited broad-spectrum antifungal activity against the growth of *Colletotrichum gloeosporioides*, *C. musae*, *Rhizoctonia solani*, and *Fusarium oxysporum* f. sp. *Niveum*, with inhibitory zones having diameters ranging from 13.90 to 17.28 mm [34]. Compounds **27** and **28** are a chromene derivative and a monoterpenoid glucoside, respectively [34]. Compounds **29** and **30** are two highly oxygenated flavonoid glycosides exhibiting potent 2,2-diphenyl-1-picrylhydrazyl radical scavenging activity, with IC50 values of 12.0 and 22.9 μM, respectively [35].

### 2.2. Chemical Components of E. chinense and Their Biological Activities

**Figure 3:**
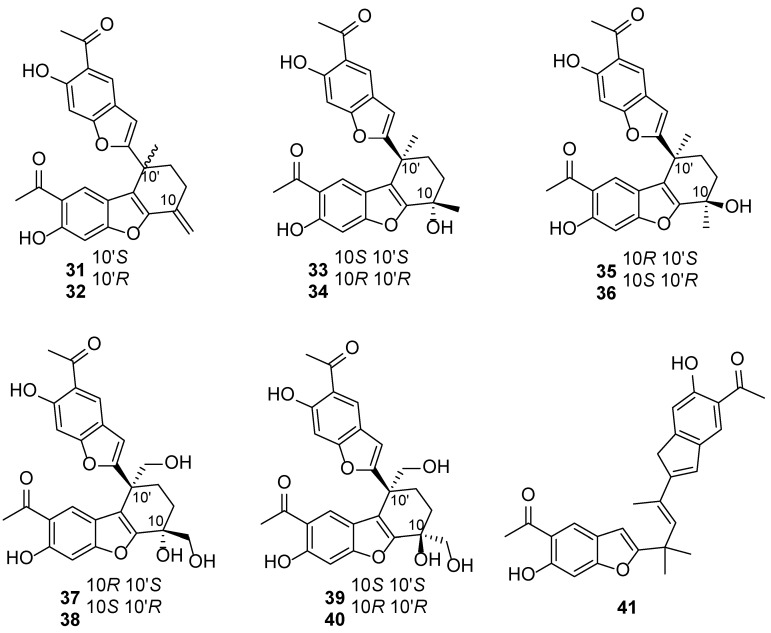

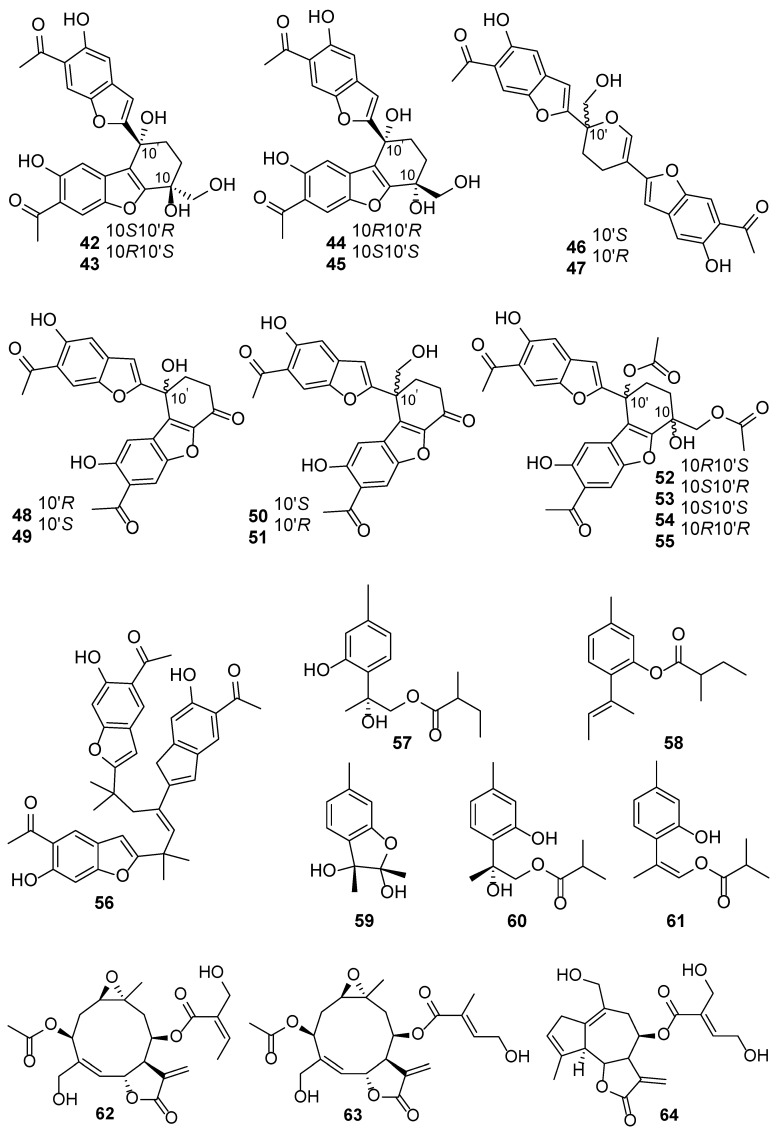

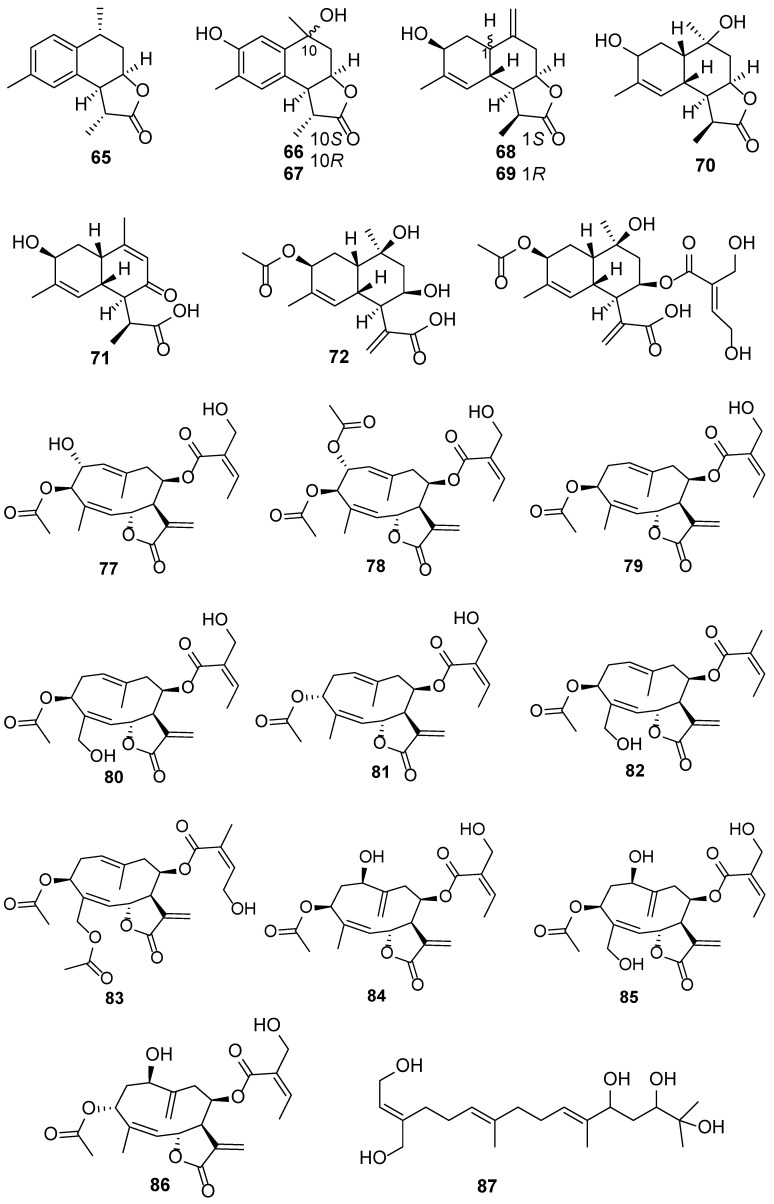
The chemical structures isolated from *E. chinense*.

*E. chinense* is used as Chinese medicine in the Tujia and Miao minorities of China. The leaves of this plant are also termed “Liu-Yue-Xue” and are used as a folk medicine for cold prevention and treatment. Its roots are widely used as a traditional Chinese medicinal material “Tu-Niu-Xi” and it has a long history of medicinal applications, because of its various pharmacological activities, such as heat-clearing, anticancer, anti-inflammatory, and antiviral activities. It is especially used as a well-known drug for the treatment of diphtheria in Guangdong Province, China [36,37]. Consequently, chemical investigations on *E. chinense* have predominantly focused on its roots to discover active components. In summary, 57 chemical constituents (**31**–**87**) were found in different parts of *E. chinense* (Table 2), namely 26 benzofuran oligomers, 25 sesquiterpenoids, 5 thymol derivatives, and 1 diterpenoid [36,37,38,39,40,41,42,43]. Of note, its roots are chiefly composed of benzofuran oligomers and thymol derivatives, whereas sesquiterpenoids are dominant in the aboveground parts (Figure 3). Compounds **31**–**56** are benzofuran dimers and trimers and are isolated from the roots. Of them, compounds **31**–**45**, **50** and **51**, as well as **54**–**56** displayed inconspicuous activities in in vitro antiviral, anti-inflammatory, and cytotoxic assays [40,41,42]. Compounds **46**–**49**, **52**, and **53** exhibited promising inhibitory effects on NO production, with IC50 values of 6.42, 6.29, and 16.03 μM, respectively [38]. Compounds **57**–**61** are thymol derivatives and are isolated from the roots [39,40]. Compound **59** exhibited moderate inhibitory effects on NO production, with the inhibition rate reaching 23.08% at 50 μM [39]. Compound **60** displayed marked cytotoxic activities against human nasopharyngeal carcinoma cells (CNE 2), human cervical cancer cells (Caski), and human gastric cancer cells (HGC-27), with IC50 values of 4.2, 11.9, and 7.3 μM, respectively [40]. Compounds **62**–**86** are sesquiterpenoids, namely 10 germacrane-type and 2 guaiane-type, and are isolated from the aerial parts of the plant [37,41,42,43]. Compounds **62** and **63** exhibited moderate cytotoxic activities against human breast cancer cells (MDA-MB-231) and human hepatocellular carcinoma cells (HepG2), with IC50 values ranging from 3.1 to 9.3 μM [37]. Compounds **79**–**81** exhibited cytotoxicity against MDA-MB-231 and HepG2, with IC50 values of 0.8–7.6 μM [43]. Compound **87** is an acyclic diterpenoid. Usually, a diterpenoid is rarely found in the genus *Eupatorium* [42].

### 2.3. Chemical Components of E. fortunei and Their Biological Activities

**Figure 4:**
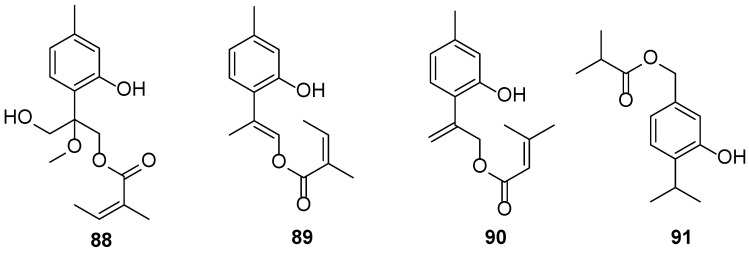

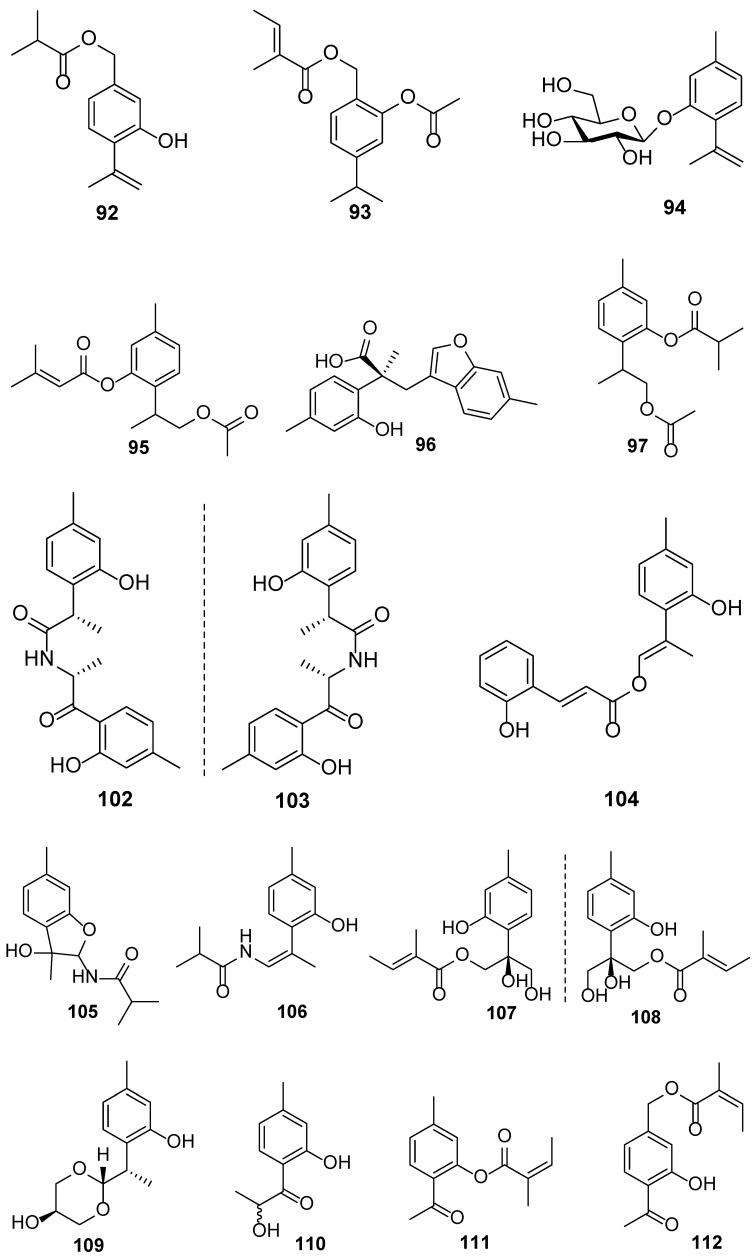

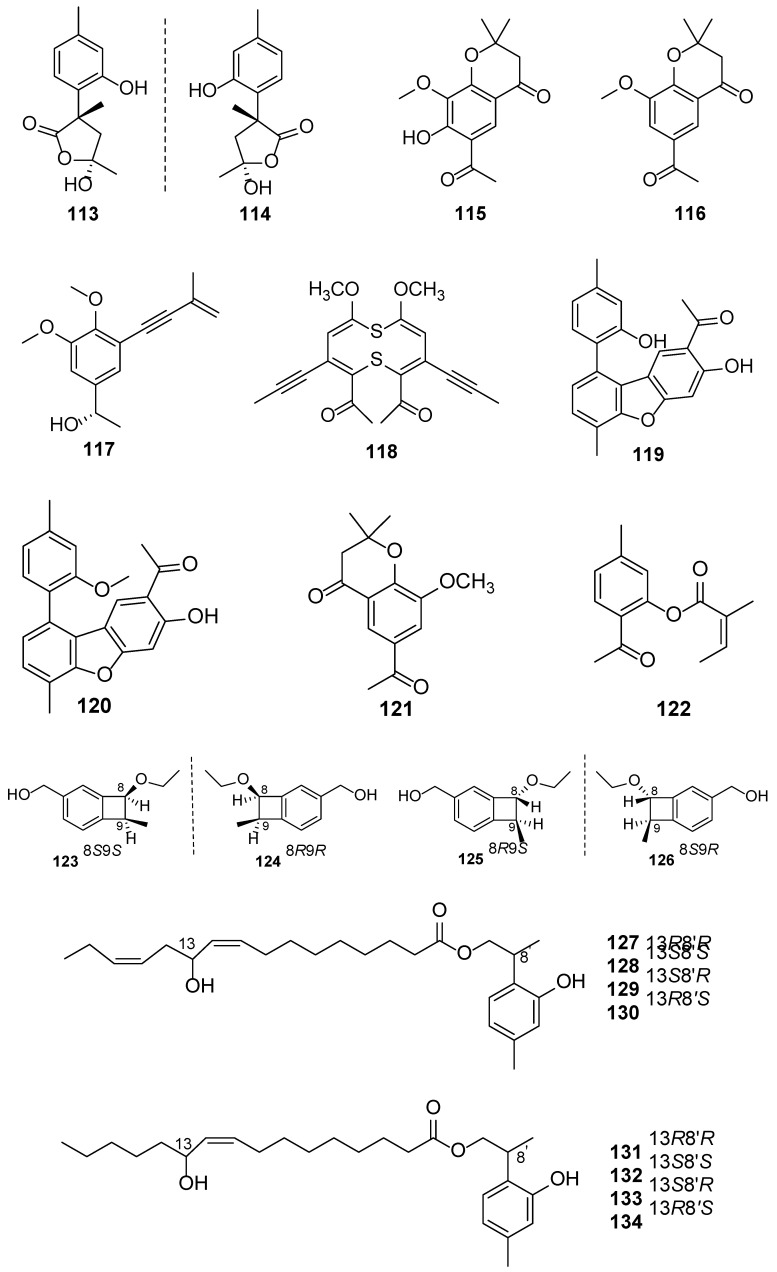

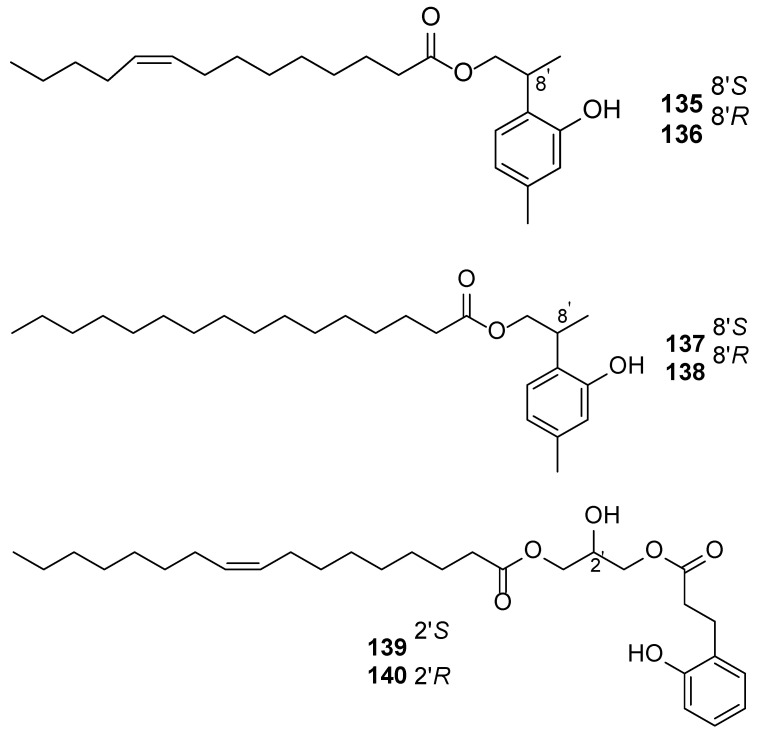
The chemical structures isolated from *E. fortunei*.

*E. fortunei* Turcz. is a perennial herb that primarily grows in the subtropical and warm temperate regions of China. Being a common aromatic and medicinal species with over 2000 years of utilization, it is widely cultivated in most eastern provinces of China. This herb has the function of removing dampness and summer heat from the body. From a modern scientific perspective, some medical symptoms relieved using this herb are partially related to inflammation. The National Health Commission of China has also incorporated this plant into the list of herbal species that can be used as additives to functional foods [44,45,46,47,48,49]. In total, 53 compounds (**88**–**140**) are isolated from the aerial parts of *E. fortunei* (Table 3), namely 27 thymol derivatives (**88**–**114**), 4 acetophenones (**115**–**118**), 2 benzofuran derivatives (**119**–**120**), 1 chromanone (**121**), 1 dithiecine (**122**), 4 monoterpenoids (**123**–**126**), and 14 fatty acid derivatives (**127**–**140**) (Figure 4) [44,45,46,47,48,49,50,51,52]. Compounds **89** and **90** exhibited cytotoxicity against MCF-7, HeLa, human lung cancer cells (A549), and HepG-2, with IC50 values of 6.24–11.96 μM [45]. Compound **105** displayed moderate activity, with an IC50 value of 24.27 μM [49]. Compound **119** showed potent cytotoxicity against A549 and MCF-7, with IC50 values of 5.95 and 5.32 µM, respectively [50]. Compounds **123**–**126** showed promising inhibitory effects on NO production, with the inhibition rate reaching 68.9%, 67.4%, 62.6%, and 65.1%, respectively, at 10 µM [51].

### 2.4. Chemical Components of E. heterophyllum and Their Biological Activities

**Figure 5:**
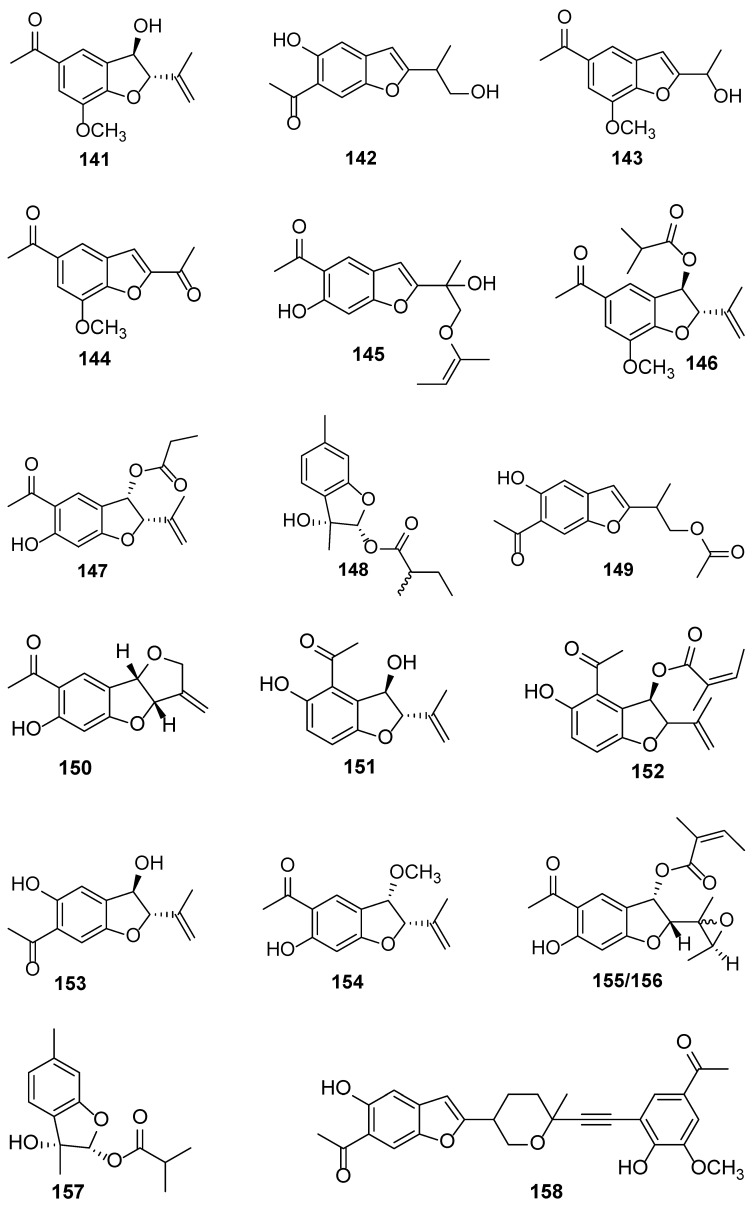

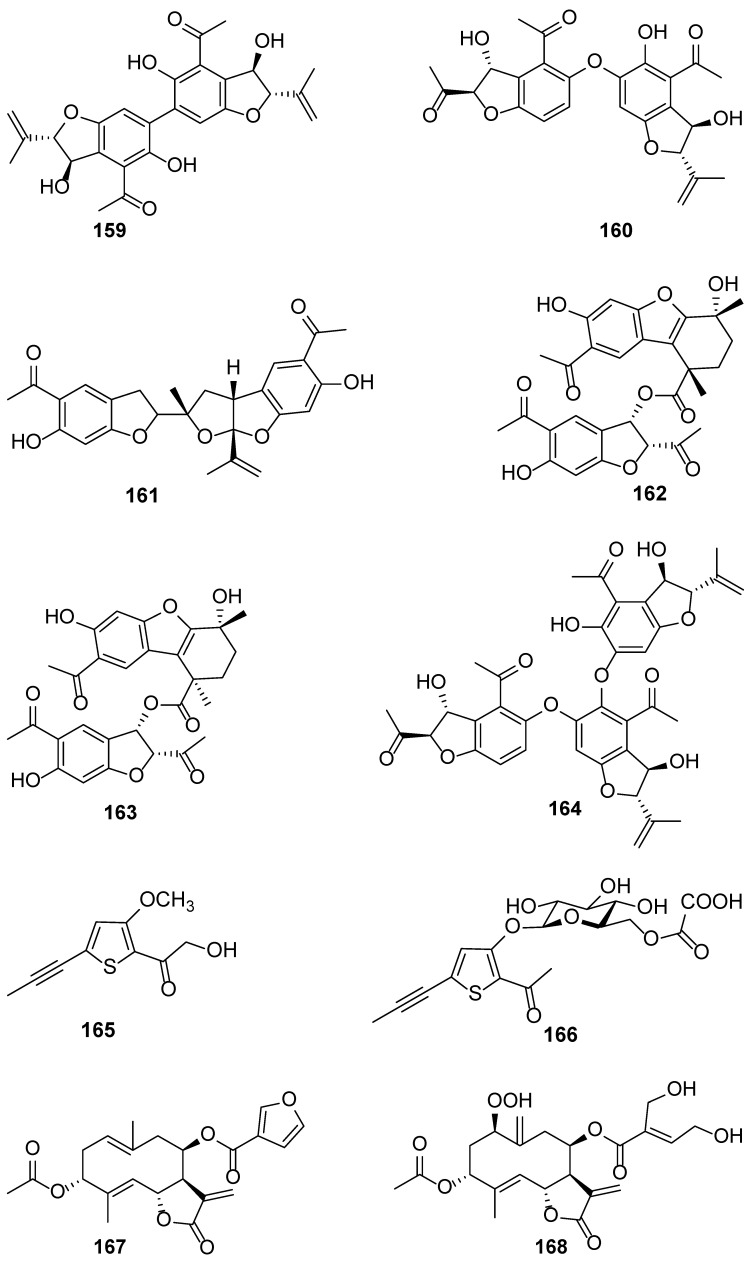

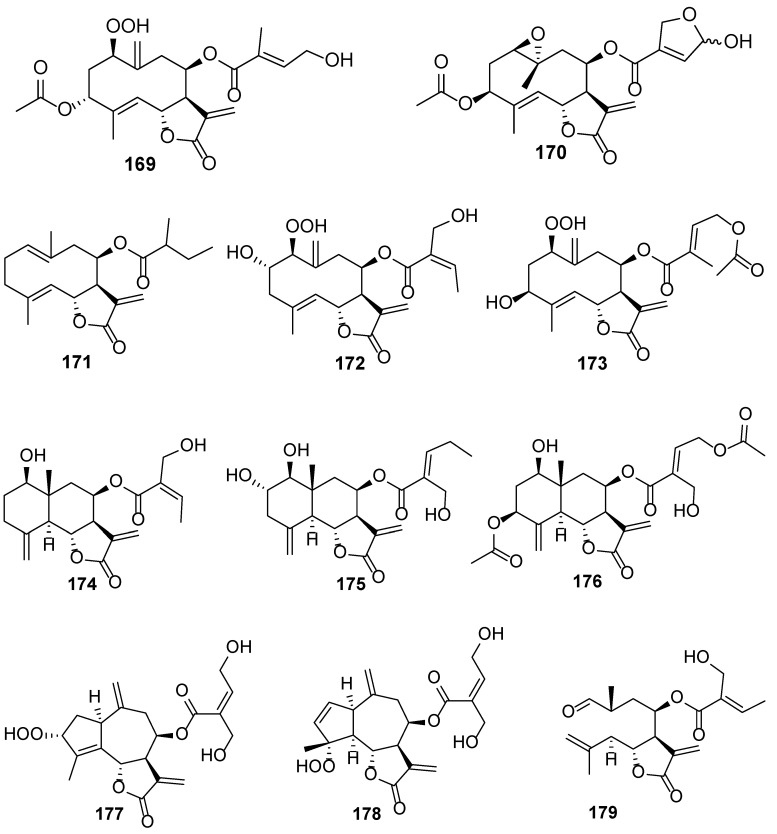
The chemical structures isolated from *E. heterophyllum*.

*E. heterophyllum* DC. is a species endemic to China and is widely distributed in the grasslands and forest areas of the Hengduan Mountains and surrounding areas, at an altitude of 1700–3000 m. In Chinese folk medicine, the stems and whole plants of this species have been used to treat various injuries and trauma [53]. However, phytochemical studies are very limited in this plant. The research on the chemical compositions of this plant species has only begun recently and the research is relatively concentrated. Therefore, this is also the first review reporting the chemical compositions of this plant (Table 4). Compounds **141**–**179** are isolated and characterized from the roots and leaves of *E. heterophyllum* (Figure 5) [53,54,55,56]. Compounds **141**–**166** are benzofuran and thiophene derivatives isolated from the roots of *E. heterophyllum* [53,54,55]. Compounds **167**–**179** are sesquiterpenoids and are isolated from the leaves of this plant [56]. Unfortunately, none of the aforementioned compounds have been evaluated for any activity. Therefore, in terms of chemical structures, the discovered compounds have a large polarity. In fact, excavation of the medium to lower polarity compounds of this plant may be continued and discoveries may happen.

### 2.5. Chemical Components of E. lindleyanum and Their Biological Activities

**Figure 6:**
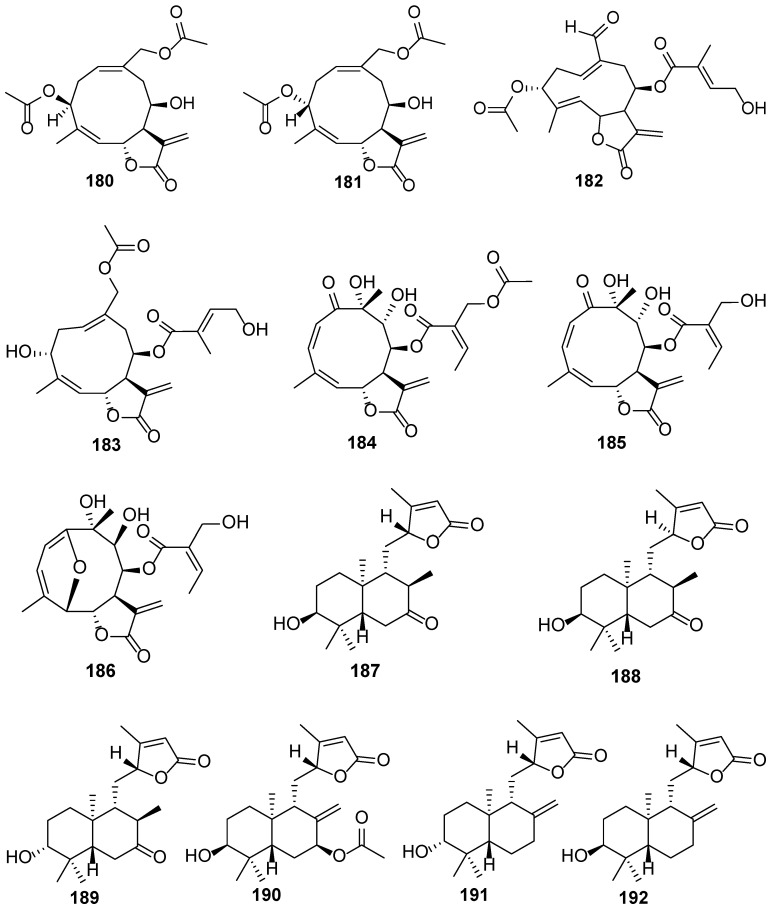
The chemical structures isolated from *E. lindleyanum*, *E. macrocephalum*, and *E. obtusissmum*.

*E. lindleyanum*, referred to as “Ye-Ma-Zhui” by the local Chinese population, is used for tracheitis and cough treatment and has a bitter, acerbic taste. Compounds **180**–**183** are sesquiterpenoids isolated from *E. lindleyanum* (Table 5 and Figure 6) [57,58,59]. Compounds **180** and **181** displayed excellent anti-inflammatory activities by lowering tumor necrosis factor-*α* and interleukin 6 levels in lipopolysaccharide-stimulated murine macrophage RAW 264.7 cells (*p* < 0.001) [57]. Compound **182** can dramatically attenuate NO secretion at 7.5 μM [58].

### 2.6. Chemical Components of E. macrocephalum and Their Biological Activities

*E. macrocephalum* Less. is a perennial herb widely distributed in the New World, from Mexico to Argentina. It is described as an invader of grasslands, wetlands, and roadsides in several provinces of South Africa. However, it is used in Paraguayan folk medicine as an anti-inflammatory and sedative agent and for the treatment of cardiac diseases [60]. Compounds **184**–**186** are three undescribed germacranolide sesquiterpenoids isolated from the aerial parts of *E. macrocephalum* (Table 5 and Figure 6). Of them, compounds **184** and **185** displayed moderate-to-potent cytotoxicity against nine human cancer cell lines, namely human glioma cells (U251), human melanoma cells (UACC-62), MCF-7, human multiple-drug resistant breast cancer cells (NCI/ADR-RES), renal clear cell adenocarcinoma cells (786–0), non-small cell lung cancer cells (NCI–H460), human ovarian cancer cells (OVCAR-3), human colon cancer cells (HT-29), and human erythroleukemia cells (K562), with IC50 values of 0.576–6.37 μM [60].

### 2.7. Chemical Components of E. obtusissmum and Their Biological Activities

*E. obtusissmum* P. DC. is an uncommon and narrowly distributed species in *Eupatorium*. This is an endemic plant from the island of Hispaniola [61]. Therefore, only one report is available on the chemical composition of *E. obtusissmum*. Compounds **187**–**192** are six *ent*-labdane diterpenoids and are isolated from the aerial parts of the plant (Table 5 and Figure 6). No compound displayed conspicuous cytotoxicity against A549, human breast carcinoma cells (HBL-100), HeLa, human lung tumor cells (SW1573), human breast cancer cells (T-47D), and human colorectal cancer cells (WiDr) [61].

## 3. Conclusions

This review discusses the recent discoveries of new compounds isolated and identified from seven plant species belonging to *Eupatorium*, since 2015; then, they were categorized according to the plant species. Notably, results of phytochemical investigations on *E. heterophyllum* and *E. obtusissmum* have recently been reported. Consequently, this is the first review of these two plant species. Although several compounds have shown anticancer, antibacterial, and anti-inflammatory effects, more compounds exhibited no significant activities and even some new compounds displayed no activity in bioactivity assays. However, if activity assays are continued in vivo or in vitro for these new compounds, we believe that more positive results may be obtained. By providing an essential reference and fresh insights, we hope this review of the recent research progress on the chemical constituents of *Eupatorium* can support and inspire researchers engaged in studies on natural products and their biological properties.

## Figures and Tables

**Table 1:** Chemical constituents (**1**–**30**) from the plant *E. adenophorum*.

No.	Plant Source	Compound Name	Structure Classification	Extraction Method	Type of Bioactivity Evaluation	Ref.
**1**	*E. adenophorum*	Eupatorid A	Sesquiterpenoid	Petroleum ether at room temperature	Anti-inflammatory, antibacterial, and cytotoxic	[23]
**2**	*E. adenophorum*	Eupatorester A	Sesquiterpenoid	Petroleum ether at room temperature	Anti-inflammatory, antibacterial, and cytotoxic	[23]
**3**	*E. adenophorum*	Eupatorester B	Sesquiterpenoid	Petroleum ether at room temperature	Anti-inflammatory, antibacterial, and cytotoxic	[23]
**4**	*E. adenophorum*	Eupatorester C	Sesquiterpenoid	Petroleum ether at room temperature	Anti-inflammatory, antibacterial, and cytotoxic	[23]
**5**	*E. adenophorum*	Adenophorone	Sesquiterpenoid	Reflux with ethyl acetate	Neuroprotective	[24]
**6**	*E. adenophorum*	Eupatorione A	Sesquiterpenoid	Petroleum ether at room temperature	Anti-inflammatory	[25]
**7**	*E. adenophorum*	Dihyroeupatorid A	Sesquiterpenoid	Petroleum ether at room temperature	Anti-inflammatory and cytotoxic	[26]
**8**	*E. adenophorum*	(5*S*, 6*S*, 7*R*, 9*R*, 10*S*)-7-Hydroxyageraphorone	Sesquiterpenoid	Petroleum ether at room temperature	Anti-inflammatory and cytotoxic	[26]
**9**	*E. adenophorum*	Adenophorone	Sesquiterpenoid	Methanol at room temperature	*α*-glycosidase and AChE inhibitory	[27]
**10**	*E. adenophorum*	Eupatorinone A	Sesquiterpenoid	95% ethanol at room temperature	Cytotoxic and antidiabetic	[28]
**11**	*E. adenophorum*	Eupatorinone B	Sesquiterpenoid	95% ethanol at room temperature	Cytotoxic and antidiabetic	[28]
**12**	*E. adenophorum*	Eupatorinone C	Sesquiterpenoid	95% ethanol at room temperature	Cytotoxic and antidiabetic	[28]
**13**	*E. adenophorum*	Ageratinone A	Sesquiterpenoid	Petroleum ether at room temperature	Cytotoxic	[29]
**14**	*E. adenophorum*	Ageratinone B	Sesquiterpenoid	Petroleum ether at room temperature	Cytotoxic	[29]
**15**	*E. adenophorum*	Ageratinone C	Sesquiterpenoid	Petroleum ether at room temperature	Cytotoxic	[29]
**16**	*E. adenophorum*	Eupatorinol	Sesquiterpenoid	95% ethanol at room temperature	Cytotoxic	[30]
**17**	*E. adenophorum*	1,6-Dihydroxy-1-isopropyl-4,7-dimethyl-3,4- dihydronaphthalen-2(1H)-one	Sesquiterpenoid	95% ethanol at room temperature	Antibacterial	[31]
**18**	*E. adenophorum*	2*α*-Methoxyl-3*β*-methyl-6-(acetyl-*O*-methyl)-2,3-dihydrobenzofuran	Thymol	95% ethanol at room temperature	Antibacterial	[31]
**19**	*E. adenophorum*	7-Formyl-9-isobutyryloxy-8-hydroxythymol	Thymol	95% ethanol at room temperature	Antibacterial and cytotoxic	[32]
**20**	*E. adenophorum*	7,9-Di-isobutyryloxy-8,10-dehydrothymol	Thymol	95% ethanol at room temperature	Antibacterial and cytotoxic	[32]
**21**	*E. adenophorum*	2a-Methoxyl-3b-methyl-6-methylol-2,3-dihydrobenzofuran	Thymol	95% ethanol at room temperature	Antibacterial and cytotoxic	[32]
**22**	*E. adenophorum*	7,9-Diisobutyryloxy-8-ethoxythymol	Thymol	95% ethanol at room temperature	Antibacterial and cytotoxic	[33]
**23**	*E. adenophorum*	7-Acetoxy-8-methoxy-9-isobutyryloxythymol	Thymol	95% ethanol at room temperature	Antibacterial and cytotoxic	[33]
**24**	*E. adenophorum*	7-Hydroxy-dehydrotremetone	Benzofuran	Methanol at room temperature	Antipathogenic fungi	[34]
**25**	*E. adenophorum*	7,10,11-Trihydroxy-dehydrotremetone	Benzofuran	Methanol at room temperature	Antipathogenic fungi	[34]
**26**	*E. adenophorum*	10-oxo-7-Hydroxy-nordehydrotremetone	Benzofuran	Methanol at room temperature	Antipathogenic fungi	[34]
**27**	*E. adenophorum*	5-*β*-Glucosyl-7-demethoxy-encecalin	Chromene	Methanol at room temperature	Antipathogenic fungi	[34]
**28**	*E. adenophorum*	8-Hydroxy-8-*β*-glucosyl-2-carene	Monoterpenoid	Methanol at room temperature	Antipathogenic fungi	[34]
**29**	*E. adenophorum*	Gossypetin-5-*O*-(6″-(*E*)-caffeoyl)-*β*-D-glucoside	Flavonoid	Reflux with 70% ethanol	Cytotoxic and antiradical	[35]
**30**	*E. adenophorum*	Herbacetin-5-*O*-(6″-(*E*)-caffeoyl)-*β*-D-glucoside	Flavonoid	Reflux with 70% ethanol	Cytotoxic and antiradical	[35]

**Table 2:** Chemical constituents (**31**–**87**) from the plant *E. chinense*.

No.	Plant Source	Compound Name	Structure Classification	Extraction Method	Type of Bioactivity Evaluation	Ref.
**31**	*E. chinense*	(+)-Dieupachinin A	Benzofuran	Reflux with 70% ethanol	Antiviral	[36]
**32**	*E. chinense*	(−)-Dieupachinin A	Benzofuran	Reflux with 70% ethanol	Antiviral	[36]
**33**	*E. chinense*	(+)-Dieupachinin B	Benzofuran	Reflux with 70% ethanol	Antiviral	[36]
**34**	*E. chinense*	(−)-Dieupachinin B	Benzofuran	Reflux with 70% ethanol	Antiviral	[36]
**35**	*E. chinense*	(+)-Dieupachinin C	Benzofuran	Reflux with 70% ethanol	Antiviral	[36]
**36**	*E. chinense*	(−)-Dieupachinin C	Benzofuran	Reflux with 70% ethanol	Antiviral	[36]
**37**	*E. chinense*	(+)-Dieupachinin D	Benzofuran	Reflux with 70% ethanol	Antiviral	[36]
**38**	*E. chinense*	(−)-Dieupachinin D	Benzofuran	Reflux with 70% ethanol	Antiviral	[36]
**39**	*E. chinense*	(+)-Dieupachinin E	Benzofuran	Reflux with 70% ethanol	Antiviral	[36]
**40**	*E. chinense*	(−)-Dieupachinin E	Benzofuran	Reflux with 70% ethanol	Antiviral	[36]
**41**	*E. chinense*	Dieupachinin F	Benzofuran	Reflux with 70% ethanol	Antiviral	[36]
**42**	*E. chinense*	(+)-Dieupachinin G	Benzofuran	95% ethanol at room temperature	Cytotoxic	[37]
**43**	*E. chinense*	(−)-Dieupachinin G	Benzofuran	95% ethanol at room temperature	Cytotoxic	[37]
**44**	*E. chinense*	(+)-Dieupachinin H	Benzofuran	95% ethanol at room temperature	Cytotoxic	[37]
**45**	*E. chinense*	(−)-Dieupachinin H	Benzofuran	95% ethanol at room temperature	Cytotoxic	[37]
**46**	*E. chinense*	(+)-Dieupachinin I	Benzofuran	95% ethanol at room temperature	Anti-inflammatory	[38]
**47**	*E. chinense*	(−)-Dieupachinin I	Benzofuran	95% ethanol at room temperature	Anti-inflammatory	[38]
**48**	*E. chinense*	(+)-Dieupachinin J	Benzofuran	95% ethanol at room temperature	Anti-inflammatory	[38]
**49**	*E. chinense*	(−)-Dieupachinin J	Benzofuran	95% ethanol at room temperature	Anti-inflammatory	[38]
**50**	*E. chinense*	(+)-Dieupachinin K	Benzofuran	95% ethanol at room temperature	Anti-inflammatory	[38]
**51**	*E. chinense*	(−)-Dieupachinin K	Benzofuran	95% ethanol at room temperature	Anti-inflammatory	[38]
**52**	*E. chinense*	(+)-Dieupachinin L	Benzofuran	95% ethanol at room temperature	Anti-inflammatory	[38]
**53**	*E. chinense*	(−)-Dieupachinin L	Benzofuran	95% ethanol at room temperature	Anti-inflammatory	[38]
**54**	*E. chinense*	(+)-Dieupachinin M	Benzofuran	95% ethanol at room temperature	Anti-inflammatory	[38]
**55**	*E. chinense*	(−)-Dieupachinin M	Benzofuran	95% ethanol at room temperature	Anti-inflammatory	[38]
**56**	*E. chinense*	Trieupachinin A	Benzofuran	Reflux with 70% ethanol	Antiviral	[36]
**57**	*E. chinense*	8*R*-hydroxy-9-methyl-butyryloxythymol	Thymol	95% ethanol at room temperature	Cytotoxic and anti-inflammatory	[39]
**58**	*E. chinense*	10-isobutyryloxy-8, 9-didehydrothymyl-isobutyrate	Thymol	95% ethanol at room temperature	Cytotoxic and anti-inflammatory	[39]
**59**	*E. chinense*	(8*R*, 9*S*)-1, 8-dimethyl-8, 9-dihydro benzofuran-8, 9-diol	Thymol	95% ethanol at room temperature	Cytotoxic and anti-inflammatory	[39]
**60**	*E. chinense*	8*R*-hydroxy-9-isobutyryloxythymol	Thymol	95% ethanol at room temperature	Cytotoxic	[40]
**61**	*E. chinense*	(Z)-8(9)-ene-9-isobutyryloxythymol	Thymol	95% ethanol at room temperature	Cytotoxic	[40]
**62**	*E. chinense*	Eupachinsin E	Sesquiterpenoid	95% ethanol at room temperature	Cytotoxic	[37]
**63**	*E. chinense*	Eupachinsin F	Sesquiterpenoid	95% ethanol at room temperature	Cytotoxic	[37]
**64**	*E. chinense*	14-Deacetylguaiaglehnin A	Sesquiterpenoid	95% ethanol at room temperature	Cytotoxic	[37]
**65**	*E. chinense*	Eupatorinolide A	Sesquiterpenoid	95% ethanol at room temperature	None	[41]
**66**	*E. chinense*	Eupatorinolide B	Sesquiterpenoid	95% ethanol at room temperature	None	[41]
**67**	*E. chinense*	Eupatorinolide C	Sesquiterpenoid	95% ethanol at room temperature	None	[41]
**68**	*E. chinense*	Eupatorinolide D	Sesquiterpenoid	95% ethanol at room temperature	None	[41]
**69**	*E. chinense*	Eupatorinolide E	Sesquiterpenoid	95% ethanol at room temperature	None	[41]
**70**	*E. chinense*	Eupatorinolide F	Sesquiterpenoid	95% ethanol at room temperature	None	[41]
**71**	*E. chinense*	Eupatorinic acid A	Sesquiterpenoid	95% ethanol at room temperature	None	[41]
**72**	*E. chinense*	Eupatorinic acid B	Sesquiterpenoid	95% ethanol at room temperature	None	[41]
**73**	*E. chinense*	Eupatorinic acid C	Sesquiterpenoid	95% ethanol at room temperature	None	[41]
**74**	*E. chinense*	Eupatorinic acid D	Sesquiterpenoid	95% ethanol at room temperature	None	[41]
**75**	*E. chinense*	Eupaguaiane A	Sesquiterpenoid	95% ethanol at room temperature	Cytotoxic	[42]
**76**	*E. chinense*	Eupaguaiane B	Sesquiterpenoid	95% ethanol at room temperature	Cytotoxic	[42]
**77**	*E. chinense*	Eupachinsin A	Sesquiterpenoid	95% ethanol at room temperature	Cytotoxic	[43]
**78**	*E. chinense*	Eupachinisin A 2-acetate	Sesquiterpenoid	95% ethanol at room temperature	Cytotoxic	[43]
**79**	*E. chinense*	Eupachinsin B	Sesquiterpenoid	95% ethanol at room temperature	Cytotoxic	[43]
**80**	*E. chinense*	3-Epi-eupachinisin B	Sesquiterpenoid	95% ethanol at room temperature	Cytotoxic	[43]
**81**	*E. chinense*	15-Hydroxyeupachinisin B	Sesquiterpenoid	95% ethanol at room temperature	Cytotoxic	[43]
**82**	*E. chinense*	Eupachinsin C	Sesquiterpenoid	95% ethanol at room temperature	Cytotoxic	[43]
**83**	*E. chinense*	4′-Hydroxyeupachinisin C 15-acetate	Sesquiterpenoid	95% ethanol at room temperature	Cytotoxic	[43]
**84**	*E. chinense*	Eupachinsin D	Sesquiterpenoid	95% ethanol at room temperature	Cytotoxic	[43]
**85**	*E. chinense*	15-Hydroxyeupachinisin D	Sesquiterpenoid	95% ethanol at room temperature	Cytotoxic	[43]
**86**	*E. chinense*	3-Epi-eupachinisin D	Sesquiterpenoid	95% ethanol at room temperature	Cytotoxic	[43]
**87**	*E. chinense*	Eupaditerpenoid A	Diterpenoid	95% ethanol at room temperature	Cytotoxic	[42]

**Table 3:** Chemical constituents (**88**–**140**) from the plant *E. fortunei*.

No.	Plant Source	Compound Name	Structure Classification	Extraction Method	Type of Bioactivity Evaluation	Ref.
**88**	*E. fortunei*	9-O-Angeloxy-10-hydroxy-8-methoxythymol	Thymol	Methanol at room temperature	None	[44]
**89**	*E. fortunei*	9-Angeloyloxy-8,9-dehydrothymol	Thymol	Refluxed with 95% ethanol	Cytotoxic	[45]
**90**	*E. fortunei*	9-(3-Methyl-2-butenoyloxy)-8,10-dehydrothymol	Thymol	Refluxed with 95% ethanol	Cytotoxic	[45]
**91**	*E. fortunei*	7-Isobutyryloxythymol	Thymol	Refluxed with 95% ethanol	Cytotoxic	[45]
**92**	*E. fortunei*	7-Isobutyryloxy-8,9-dehydrothymol	Thymol	Refluxed with 95% ethanol	Cytotoxic	[45]
**93**	*E. fortunei*	2-Acetyl-7-tigloyloxy-isothymol	Isothymol	Refluxed with 95% ethanol	Cytotoxic	[45]
**94**	*E. fortunei*	8, 9-dehydrothymol-3-*O*-*β*-glucoside	Thymol	95% ethanol at room temperature	Cytotoxic	[46]
**95**	*E. fortunei*	3-methylbut-2-enoate	Thymol	95% ethanol at room temperature	Cytotoxic	[46]
**96**	*E. fortunei*	2-(2-hydroxy-4-methylphenyl)-2-methyl-3-(5-methylbenzofuran-3-yl)propanoic acid	Thymol	Methanol at room temperature	None	[47]
**97**	*E. fortunei*	9-acetoxyl-3-isobutyroylthymol	Thymol	Methanol at room temperature	a-Glucosidase and acetylcholinesterase inhibitory	[47]
**98**	*E. fortunei*	7,8,9-trihydroxythymol	Thymol	95% ethanol at room temperature	Antibacterial	[48]
**99**	*E. fortunei*	8,10-didehydro-7,9-dihydroxythymol	Thymol	95% ethanol at room temperature	Antibacterial	[48]
**100**	*E. fortunei*	(−)-Eupafortunin A	Thymol	95% ethanol at room temperature	Antiradical and anti-inflammatory	[49]
**101**	*E. fortunei*	(+)-Eupafortunin A	Thymol	95% ethanol at room temperature	Antiradical and anti-inflammatory	[49]
**102**	*E. fortunei*	(+)-Eupafortunin B	Thymol	95% ethanol at room temperature	Antiradical and anti-inflammatory	[49]
**103**	*E. fortunei*	(−)-eupafortunin B	Thymol	95% ethanol at room temperature	Antiradical and anti-inflammatory	[49]
**104**	*E. fortunei*	Eupafortunin C	Thymol	95% ethanol at room temperature	Antiradical and anti-inflammatory	[49]
**105**	*E. fortunei*	Eupafortunin D	Thymol	95% ethanol at room temperature	Antiradical and anti-inflammatory	[49]
**106**	*E. fortunei*	Eupafortunin E	Thymol	95% ethanol at room temperature	Antiradical and anti-inflammatory	[49]
**107**	*E. fortunei*	(+)-Eupafortunin F	Thymol	95% ethanol at room temperature	Antiradical and anti-inflammatory	[49]
**108**	*E. fortunei*	(−)-Eupafortunin F	Thymol	95% ethanol at room temperature	Antiradical and anti-inflammatory	[49]
**109**	*E. fortunei*	Eupafortunin G	Thymol	95% ethanol at room temperature	Antiradical and anti-inflammatory	[49]
**110**	*E. fortunei*	Eupafortunin H	Thymol	95% ethanol at room temperature	Antiradical and anti-inflammatory	[49]
**111**	*E. fortunei*	Eupafortunin I	Thymol	95% ethanol at room temperature	Antiradical and anti-inflammatory	[49]
**112**	*E. fortunei*	Eupafortunin J	Thymol	95% ethanol at room temperature	Antiradical and anti-inflammatory	[49]
**113**	*E. fortunei*	(+)-Eupafortunin K	Thymol	95% ethanol at room temperature	Antiradical and anti-inflammatory	[49]
**114**	*E. fortunei*	(−)-Eupafortunin K	Thymol	95% ethanol at room temperature	Antiradical and anti-inflammatory	[49]
**115**	*E. fortunei*	Eupafortunin L	Acetophenone	95% ethanol at room temperature	Antiradical and anti-inflammatory	[49]
**116**	*E. fortunei*	Eupafortunin M	Acetophenone	95% ethanol at room temperature	Antiradical and anti-inflammatory	[49]
**117**	*E. fortunei*	Eupafortunin N	Acetophenone	95% ethanol at room temperature	Antiradical and anti-inflammatory	[49]
**118**	*E. fortunei*	Eupatofortunone	Acetophenone	Methanol at room temperature	Cytotoxic	[50]
**119**	*E. fortunei*	Eupatodibenzofuran A	Benzofuran	Methanol at room temperature	Cytotoxic	[50]
**120**	*E. fortunei*	Eupatodibenzofuran B	Benzofuran	Methanol at room temperature	Cytotoxic	[50]
**121**	*E. fortunei*	6-acetyl-8-methoxy-2,2-dimethylchroman-4-one	Chromanone	Methanol at room temperature	Cytotoxic	[50]
**122**	*E. fortunei*	Eupatodithiecine	Dithiecine	Methanol at room temperature	Cytotoxic	[50]
**123**	*E. fortunei*	(+)-Eupafortin A	Monoterpenoid	95% ethanol at room temperature	Anti-inflammatory	[51]
**124**	*E. fortunei*	(−)-Eupafortin A	Monoterpenoid	95% ethanol at room temperature	Anti-inflammatory	[51]
**125**	*E. fortunei*	(+)-Eupafortin B	Monoterpenoid	95% ethanol at room temperature	Anti-inflammatory	[51]
**126**	*E. fortunei*	(−)-Eupafortin B	Monoterpenoid	95% ethanol at room temperature	Anti-inflammatory	[51]
**127**	*E. fortunei*	Eupatorid A	Fatty acid	95% ethanol at room temperature	Anti-inflammatory	[52]
**128**	*E. fortunei*	Eupatorid A	Fatty acid	95% ethanol at room temperature	Anti-inflammatory	[52]
**129**	*E. fortunei*	Eupatorid B	Fatty acid	95% ethanol at room temperature	Anti-inflammatory	[52]
**130**	*E. fortunei*	Eupatorid B	Fatty acid	95% ethanol at room temperature	Anti-inflammatory	[52]
**131**	*E. fortunei*	Eupatorid C	Fatty acid	95% ethanol at room temperature	Anti-inflammatory	[52]
**132**	*E. fortunei*	Eupatorid C	Fatty acid	95% ethanol at room temperature	Anti-inflammatory	[52]
**133**	*E. fortunei*	Eupatorid D	Fatty acid	95% ethanol at room temperature	Anti-inflammatory	[52]
**134**	*E. fortunei*	Eupatorid D	Fatty acid	95% ethanol at room temperature	Anti-inflammatory	[52]
**135**	*E. fortunei*	Eupatorid E	Fatty acid	95% ethanol at room temperature	Anti-inflammatory	[52]
**136**	*E. fortunei*	Eupatorid E	Fatty acid	95% ethanol at room temperature	Anti-inflammatory	[52]
**137**	*E. fortunei*	Eupatorid F	Fatty acid	95% ethanol at room temperature	Anti-inflammatory	[52]
**138**	*E. fortunei*	Eupatorid F	Fatty acid	95% ethanol at room temperature	Anti-inflammatory	[52]
**139**	*E. fortunei*	Eupatorid G	Fatty acid	95% ethanol at room temperature	Anti-inflammatory	[52]
**140**	*E. fortunei*	Eupatorid G	Fatty acid	95% ethanol at room temperature	Anti-inflammatory	[52]

**Table 4:** Chemical constituents (**141**–**179**) from the plant *E. heterophyllum*.

No.	Plant Source	Compound Name	Structure Classification	Extraction Method	Type of Bioactivity Evaluation	Ref.
**141**	*E. heterophyllum*	Eupaheterin A	Benzofuran	Methanol at room temperature	None	[53]
**142**	*E. heterophyllum*	Eupaheterin B	Benzofuran	Methanol at room temperature	None	[53]
**143**	*E. heterophyllum*	Eupaheterin C	Benzofuran	Methanol at room temperature	None	[53]
**144**	*E. heterophyllum*	Eupaheterin D	Benzofuran	Methanol at room temperature	None	[53]
**145**	*E. heterophyllum*	Eupaheterin E	Benzofuran	Methanol at room temperature	None	[53]
**146**	*E. heterophyllum*	Eupaheterin F	Benzofuran	Methanol at room temperature	None	[53]
**147**	*E. heterophyllum*	Eupaheterin G	Benzofuran	Methanol at room temperature	None	[53]
**148**	*E. heterophyllum*	Eupaheterin H	Benzofuran	Methanol at room temperature	None	[53]
**149**	*E. heterophyllum*	Eupaheterin I	Benzofuran	Methanol at room temperature	None	[53]
**150**	*E. heterophyllum*	Eupaheterin J	Benzofuran	Methanol at room temperature	None	[53]
**151**	*E. heterophyllum*	4-Acetyl-3*β*,5-dihydroxy-2*α*-(propen-2-yl)-2,3-dihydrobenzofuran	Benzofuran	Methanol at room temperature	None	[54]
**152**	*E. heterophyllum*	4-Acetyl-3*β*-angeloyloxy-5-hydroxy-2*α*-(propen-2-yl)- 2,3-dihydrobenzofuran	Benzofuran	Methanol at room temperature	None	[54]
**153**	*E. heterophyllum*	6-Acetyl-3*β*,5-dihydroxy-2*α*-(propen-2-yl)-2,3-dihydrobenzofuran	Benzofuran	Methanol at room temperature	None	[54]
**154**	*E. heterophyllum*	5-Acetyl-6-hydroxy-3*α*-methoxyl-2*α*-(propen-2-yl)-2,3- dihydrobenzofuran	Benzofuran	Methanol at room temperature	None	[54]
**155**	*E. heterophyllum*	5-Acetyl-3*α*-angeloyloxy-6-hydroxy-2*α*-(2-methyloxiran-2-yl)-2,3-dihydrobenzofuran	Benzofuran	Methanol at room temperature	None	[54]
**156**	*E. heterophyllum*	5-Acetyl-3*α*-angeloyloxy-6-hydroxy-2*α*-(2-methyloxiran-2-yl)-2,3-dihydrobenzofuran	Benzofuran	Methanol at room temperature	None	[54]
**157**	*E. heterophyllum*	3,9*β*-Epoxy-9*α*-isobutanoyloxymentha-13,5-trien-8*α*-ol	Benzofuran	Methanol at room temperature	None	[54]
**158**	*E. heterophyllum*	Dieupaheterin A	Benzofuran	Methanol at room temperature	None	[53]
**159**	*E. heterophyllum*	Dieupaheterin B	Benzofuran	Methanol at room temperature	None	[53]
**160**	*E. heterophyllum*	Dieupaheterin C	Benzofuran	Methanol at room temperature	None	[53]
**161**	*E. heterophyllum*	Dieupaheterin D	Benzofuran	Methanol at room temperature	None	[53]
**162**	*E. heterophyllum*	Dieupaheterin E	Benzofuran	Methanol at room temperature	None	[55]
**163**	*E. heterophyllum*	Dieupaheterin F	Benzofuran	Methanol at room temperature	None	[55]
**164**	*E. heterophyllum*	Trieupaheterin A	Benzofuran	Methanol at room temperature	None	[53]
**165**	*E. heterophyllum*	2-(Hydroxyacetyl)-3-methoxy-5-(propyn-1-yl)thiophene	Thiophene	Methanol at room temperature	None	[54]
**166**	*E. heterophyllum*	2-Acetyl-3-hydroxy-5-(propyn-1-yl)thiophene-3-*O*-(6-*O*-malonyl)-*β*-glucoside	Thiophene	Methanol at room temperature	None	[54]
**167**	*E. heterophyllum*	(3*R*,6*R*,7*R*,8*R*)-(4*Z*)-3*α*-acetoxy-8*β*-(3-furoyloxy)germacra-1(10),4,11(13)-trien-(12,6α)-olide	Sesquiterpenoid	Methanol at room temperature	None	[56]
**168**	*E. heterophyllum*	(4*Z*)-3*α*-acetoxy-8*β*-(4′,5′ -dihydroxytigloyloxy)-1β-hydroperoxygermacra-4,10(14),11(13)-trien-(12,6α)-olide	Sesquiterpenoid	Methanol at room temperature	None	[56]
**169**	*E. heterophyllum*	5′-deoxy-(4Z)-3α-acetoxy-8β-(4′,5′ -dihydroxytigloyloxy)-1β-hydroperoxygermacra-4,10(14),11(13)-trien-(12,6α)-olide	Sesquiterpenoid	Methanol at room temperature	None	[56]
**170**	*E. heterophyllum*	(4Z)-3*β*-acetoxy-1*β*,10α-epoxy-8*β*-(4′,5-epoxy-4′-hydroxytigloyloxy)germacra-4,11(13)-dien-(12,6*α*)-olide	Sesquiterpenoid	Methanol at room temperature	None	[56]
**171**	*E. heterophyllum*	8*β*-(2′-methylbutanoyloxy)germacra-1(10),4,11(13)-trien-(12,6*α*)-olide	Sesquiterpenoid	Methanol at room temperature	None	[56]
**172**	*E. heterophyllum*	1*β*-hydroperoxy-2*α*-hydroxy-8*β*-(5′-hydroxyangeloyloxy)germacra-4,10(14),11(13)-trien-(12,6*α*)-olide	Sesquiterpenoid	Methanol at room temperature	None	[56]
**173**	*E. heterophyllum*	8β-(4′-acetoxytigloyloxy)-1*β*-hydroperoxy-3*β*-hydroxygermacra-4,10(14),11(13)-trien-(12,6*α*)-olide	Sesquiterpenoid	Methanol at room temperature	None	[56]
**174**	*E. heterophyllum*	1*β*-hydroxy-8*β*-(5′-hydroxyangeloyloxy)eudesma-4(15),11(13)-dien-(12,6*α*)-olide	Sesquiterpenoid	Methanol at room temperature	None	[56]
**175**	*E. heterophyllum*	1*β*,2*α*-dihydroxy-8*β*-(5′-hydroxyangeloyloxy)eudesma-4(15),11(13)-dien- (12,6*α*)-olide	Sesquiterpenoid	Methanol at room temperature	None	[56]
**176**	*E. heterophyllum*		Sesquiterpenoid	Methanol at room temperature	None	[56]
**177**	*E. heterophyllum*	8*β*-(4′,5′-dihydroxytigloyloxy)-3α-hydroperoxyguaia-4,10(14),11(13)-trien-(12,6*α*)-olide	Sesquiterpenoid	Methanol at room temperature	None	[56]
**178**	*E. heterophyllum*		Sesquiterpenoid	Methanol at room temperature	None	[56]
**179**	*E. heterophyllum*	8*β*-(5′-hydroxyangeloyloxy)-1-oxo-2-norelema-3,11(13)-dien-(12,6*α*)-olid	Sesquiterpenoid	Methanol at room temperature	None	[56]

Compounds **141**–**150** and **158**–**164** were not named in the original article, but were named by the author for ease of reading.

**Table 5:** Chemical constituents from the plants *E. lindleyanum* (**180**–**183**), *E. macrocephalum* (**184**–**186**), and *E. obtusissmum* (**187**–**192**).

No.	Plant Source	Compound Name	Structure Classification	Extraction Method	Type of Bioactivity Evaluation	Ref.
**180**	*E. lindleyanum*	Eupalinolide L	Sesquiterpenoid	Boiling water	Anti-inflammatory	[57]
**181**	*E. lindleyanum*	Eupalinolide M	Sesquiterpenoid	Boiling water	Anti-inflammatory	[57]
**182**	*E. lindleyanum*	Eupalinolide N	Sesquiterpenoid	Refluxed with 90% ethanol	Anti-inflammatory	[58]
**183**	*E. lindleyanum*	Eupalinolide O	Sesquiterpenoid	95% ethanol at room temperature	Cytotoxic	[59]
**184**	*E. macrocephalum*	Macrocephalide A	Sesquiterpenoid	Methanol at room temperature	Cytotoxic	[60]
**185**	*E. macrocephalum*	Macrocephalide B	Sesquiterpenoid	Methanol at room temperature	Cytotoxic	[60]
**186**	*E. macrocephalum*	Macrocephalide C	Sesquiterpenoid	Methanol at room temperature	Cytotoxic	[60]
**187**	*E. obtusissmum*	Uasdlabdane A	Diterpenoid	95% ethanol at room temperature	Cytotoxic	[61]
**188**	*E. obtusissmum*	Uasdlabdane B	Diterpenoid	95% ethanol at room temperature	Cytotoxic	[61]
**189**	*E. obtusissmum*	Uasdlabdane C	Diterpenoid	95% ethanol at room temperature	Cytotoxic	[61]
**190**	*E. obtusissmum*	Uasdlabdane D	Diterpenoid	95% ethanol at room temperature	Cytotoxic	[61]
**191**	*E. obtusissmum*	Uasdlabdane E	Diterpenoid	95% ethanol at room temperature	Cytotoxic	[61]
**192**	*E. obtusissmum*	Uasdlabdane F	Diterpenoid	95% ethanol at room temperature	Cytotoxic	[61]

## Data Availability

No data were used for the research described in the article.
